# Stature estimation by semi-automatic measurements of 3D CT images of the femur

**DOI:** 10.1007/s00414-022-02921-y

**Published:** 2022-12-07

**Authors:** Kei Kira, Fumiko Chiba, Yohsuke Makino, Suguru Torimitsu, Rutsuko Yamaguchi, Shigeki Tsuneya, Ayumi Motomura, Maiko Yoshida, Naoki Saitoh, Go Inokuchi, Yumi Hoshioka, Hisako Saitoh, Daisuke Yajima, Hirotaro Iwase

**Affiliations:** 1 Department of Forensic Medicine, Graduate School of Medicine, The University of Tokyo, 7-3-1 Hongo, Bunkyo-Ku, Tokyo, 113-8654 Japan; 2 Department of Legal Medicine, Graduate School of Medicine, Chiba University, 1-8-1 Inohana, Chuo-Ku, Chiba, Chiba Prefecture 260-8670 Japan; 3 Department of Forensic Medicine, School of Medicine, International University of Health and Welfare, 4-3 Kozunomori, Narita, 286-8686 Japan

**Keywords:** Stature estimation, Femur, Computed tomography, Artificial intelligence, Semi-automatic measurement

## Abstract

Stature estimation is one of the most basic and important methods of personal identification. The long bones of the limbs provide the most accurate stature estimation, with the femur being one of the most useful. In all the previously reported methods of stature estimation using computed tomography (CT) images of the femur, laborious manual measurement was necessary. A semi-automatic bone measuring method can simplify this process, so we firstly reported a stature estimation process using semi-automatic bone measurement software equipped with artificial intelligence. Multiple measurements of femurs of adult Japanese cadavers were performed using automatic three-dimensional reconstructed CT images of femurs. After manually setting four points on the femur, an automatic measurement was acquired. The relationships between stature and five femoral measurements, with acceptable intraobserver and interobserver errors, were analyzed with single regression analysis using the standard error of the estimate (SEE) and the coefficient of determination (*R*^2^). The maximum length of the femur (MLF) provided the lowest SEE and the highest *R*^2^; the SEE and *R*^2^ in all cadavers, males and females, respectively, were 3.913 cm (*R*^2^ = 0.842), 3.664 cm (*R*^2^ = 0.705), and 3.456 cm (*R*^2^ = 0.686) for MLF on the right femur, and 3.837 cm (*R*^2^ = 0.848), 3.667 cm (*R*^2^ = 0.705), and 3.384 cm (*R*^2^ = 0.699) for MLF on the left femur. These results were non-inferior to those of previous reports regarding stature estimation using the MLF. Stature estimation with this simple and time-saving method would be useful in forensic medical practice.

**Supplementary Information:**

The online version contains supplementary material available at 10.1007/s00414-022-02921-y.

## Introduction

Stature estimation is one of the most important and basic methods for individual identification as well as for sex and age estimation [1–10]. Recent forensic anthropology reports have described sex, weight, and age estimation using computed tomographic (CT) images of bones [11–22]. Regarding stature estimation, the long bones of the limbs provide the most accurate stature estimation over a wide age range in studies conducted on different races. Among them, the femur is reported as one of the most useful for stature estimation [4, 10, 23–30].

Conventionally, the femur is measured using an osteometric board, which is placed on a horizontal plane [31–35]. In recent reports, the femur was measured using X-ray photography [36–38]. Some reports have provided stature estimation using CT images of the femur [39–44], and researchers in these studies manually measured the femur on CT images for estimation. However, manual measurement requires a certain level of technical proficiency and can be affected by the performance of the measurer. Thus, using a simpler measurement method than the manual method may provide benefits such as reduction of time and effort required for measurement and prevention of unintentional measurement errors. Herein, we created three-dimensional (3D) reconstructed images from postmortem CT images and measured the femur using a semi-automatic measurement software, with the aim of providing new stature estimation formulae based on these semi-automatic measurements.

## Materials and methods

This study included 300 cadavers of known sex and age over 18 that underwent whole-body postmortem CT imaging and subsequent forensic autopsy at the forensic medicine departments at Chiba University and the University of Tokyo in Japan between October 2016 and October 2020. Cadavers with severe decomposition, burn injuries, congenital malformations, postoperative changes, missing parts, femoral fractures, severe deformation of the vertebral bodies, and severe trauma to the head, neck, trunk, or lower limbs were excluded because such conditions have possible effects on the condition of the femur or stature. We included the cadavers of 150 males (10–20 years, *n* = 1; 21–30 years, *n* = 21; 31–40 years, *n* = 19; 41–50 years, *n* = 37; 51–60 years, *n* = 38; 61–70 years, *n* = 20; 71–80 years, *n* = 12; 81–90 years, *n* = 2) and 150 females (10–20 years, *n* = 13; 21–30 years, *n* = 12; 31–40 years, *n* = 28; 41–50 years, *n* = 24; 51–60 years, *n* = 21; 61–70 years, *n* = 18; 71–80 years, *n* = 20; 81–90 years, *n* = 14). Cadaver stature was measured in the supine position before autopsy using a measuring tape or a ruler. The adjusted stature (AS) was calculated by subtracting 2.0 cm from the measured stature to obtain an estimate of the living stature according to previous studies [45–48].

At Chiba University, postmortem CT was performed using a 64-row detector CT system (Supria Grande; Fujifilm Healthcare Corporation, Tokyo, Japan), and the scanning protocol was as follows: tube voltage, 120 kV; tube current, 250 mA; scan time, 0.75 s; collimation, 0.625 mm. The slice thickness, reconstruction interval, and field of view during image reconstruction were 1.0, 0.725, and 500 mm, respectively. At the University of Tokyo, postmortem CT was performed using a 16-row detector CT system (ECLOS; Fujifilm Healthcare Corporation), and the scanning protocol was as follows: tube voltage, 120 kV; tube current, 200 mA; scan time, 1 s; collimation, 1.25 mm. The slice thickness, reconstruction interval, and field of view during image reconstruction were 1.25, 1.25, and 500 mm, respectively.

**Fig. 1:**
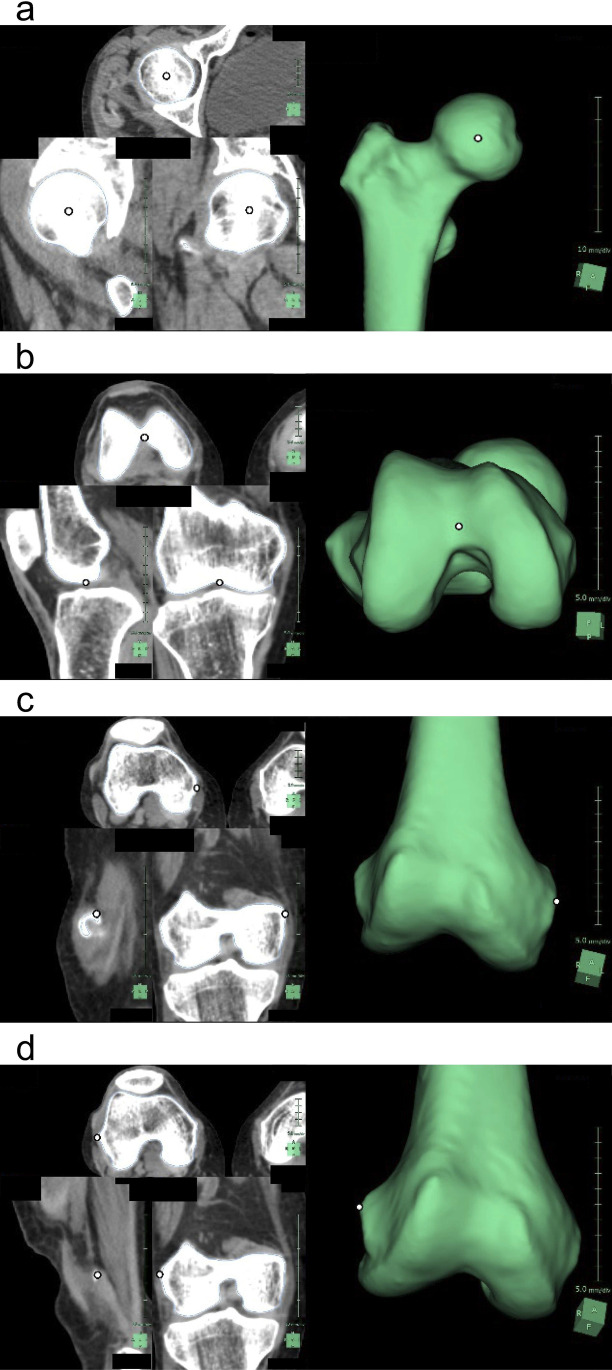
Four points where manual marking are necessary (each picture shows one point in the horizontal, coronal, and sagittal planes and the three-dimensional reconstruction of the computed tomography images). **a** Center of the femoral head: the central point of the femoral head. **b** Intercondylar notch: posterior 1/4 point on the midline of the recess located between the medial and lateral condyles on the bottom surface of the lower end of the femur. **c** Medial epicondyle: the most medial point of the medial condyle. **d** Lateral epicondyle: the most lateral point of the lateral condyle

**Fig. 2:**
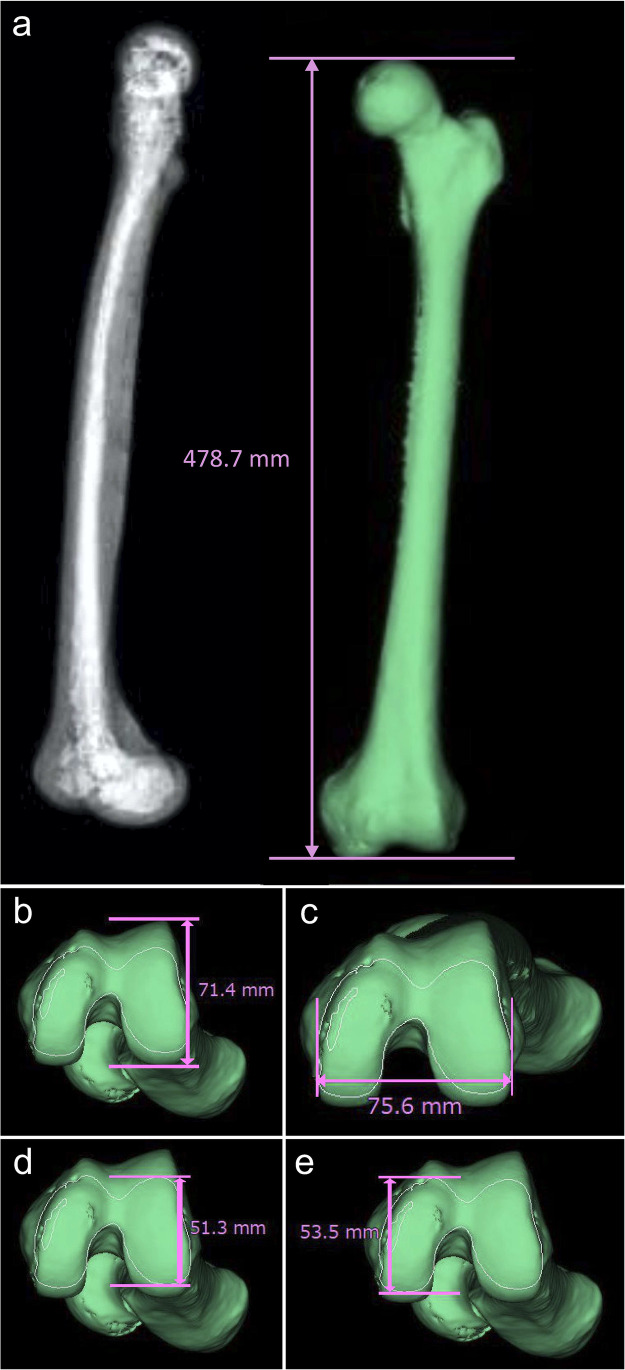
Five measurements with acceptable intraobserver and interobserver errors. **a** Maximum length of the femur (MLF). **b** Lateral anterior–posterior length (LAP). **c** Cross-section medial–lateral width (C-ML). **d** C-lateral anterior–posterior length (C-LAP). **e** C-medial anterior–posterior length (C-MAP)

Image data were processed on a workstation (Synapse Vincent; Fujifilm Medical), and a semi-automatic application was used to measure the femur. Just after launching, this application automatically recognizes the femur and displays it as a reconstructed 3D image. If it contains other structures, such as calcified blood vessels or cartilage, manual adjustments are necessary. After confirmation that the reconstruction is appropriate, the bone surface information is automatically extracted with a single click. By manually marking the four points—the center of the femoral head, intercondylar notch (ICN), medial epicondyle, and lateral epicondyle—on the model (Fig. 1), 41 measurements are automatically calculated and displayed (Table 1). The time required from manual marking to displaying the results was approximately 40 s. Using the results of each cadaver, the average values of the right and left femurs were also calculated (Fig. 2).

**Table 1:** Definition of measurements

Measurement	Abbreviation	Definition
Maximum length of the femur	MLF	Distance between the plane tangent to the lowest points of the medial and lateral condyles of the femur (referred to as plane α) and the plane parallel to plane α that is tangent to the upper end of the femur
Valuation angle	VA	Angle formed by the MA and the line segment connecting the center of the femoral diaphysis and the ICN when the femur is observed from the ventral side
Lordosis angle	LA	Angle formed by the MA and the line segment connecting the center of femoral diaphysis and the ICN when the femur is observed from the lateral side
Lateral anterior–posterior length	LAP	Distance between the two lines when lines parallel to the SEA are drawn to pass through the anterior and posterior ends of the lateral epicondyle when the femur is observed from the bottom side
Medial anterior–posterior length	MAP	Distance between the two lines when lines parallel to the SEA are drawn to pass through the anterior and posterior ends of the medial epicondyle when the femur is observed from the bottom side
Partial lateral anterior–posterior length	P-LAP	Distance between the two lines when lines parallel to the SEA is drawn to pass through the rearmost end of the lateral condyle and the posterior end of the intercondylar fossa when the femur is observed from the bottom side
Partial medial anterior–posterior length	P-MAP	Distance between the two lines when lines parallel to the SEA is drawn to pass through the rearmost end of the medial condyle and the posterior end of the intercondylar fossa when the femur is observed from the bottom side
Lateral distal resection amount	LRA	Distance between two lines when straight lines parallel to SEA are drawn through the lower end of the lateral condyle and the ICN when observed facing the plane created by SEA and MA (referred to as plane β)
Medial distal resection amount	MRA	Distance between two lines when straight lines parallel to SEA are drawn through the lower end of the medial condyle and the ICN when observed facing plane β
Cross-section partial lateral anterior–posterior length	C-P-LAP	Distance on CS A′ (which is a cross section that is orthogonal to plane β and approximates a horizontal section at a height that passes through the ICN) between the SEA and the straight line parallel to the SEA drawn so as to pass through the posterior end of the lateral condyle
Cross-section partial medial anterior–posterior length	C-P-MAP	Distance on CS A′ between the SEA and the straight line parallel to the SEA drawn so as to pass through the posterior end of the medial condyle
Posterior condyle axis angle	PCA-angle	Angle formed by the SEA and a straight line passing through the posterior ends of the medial and lateral condyles when the femur is observed from the bottom
Cross-section lateral-middle length	C-LML	Distance between the two lines when straight lines perpendicular to the SEA are drawn so as to pass through the lateral end of CS A′ and the ICN
Cross-section medial-middle length	C-MML	Distance between the two lines when straight lines perpendicular to the SEA are drawn so as to pass through the medial end of CS A′ and the ICN
Cross-section medial–lateral width	C-ML	Distance between the two lines when straight lines perpendicular to the SEA are drawn so as to pass through the medial end of CS A′ and the lateral end of CS A′
Lateral distal anterior angle	LDA-angle	Angle formed on CS A′ by the straight line orthogonal to the SEA and line passing through points A and B, which are the intersections of a straight line parallel to the SEA through ICN and the SEA with the outermost side of CS A′
Medial distal anterior angle	MDA-angle	Angle formed on CS A′ by the straight line orthogonal to the SEA and the line passing through points C and D, which are the intersections of a straight line parallel to the SEA through the ICN and the SEA with the innermost side of CS A′
Medial distal radius of the curvature	MDAC	Radiation of the curvature of point C on CS A′
Joint line angle	JL-angle	Angle formed by the line parallel to the SEA passing through the ICN and the straight line passing through the lower end of the lateral condyle and that of the medial condyle when the femur is observed from the ventral side
Medial superior-inferior length	MSI	Length from the top to the bottom of CS B′, which is a cross section formed on the medial condyle that is perpendicular to CS A′ and passes through the lateral and medial posterior ends of CS A′
Lateral superior-inferior length	LSI	Length from top to bottom of CS C′, which is a cross section formed on the lateral condyle so as to be perpendicular to CS A′ and passes through the lateral and medial posterior ends of CS A′
Medial condyle width	MCW	Length from the medial end to the lateral end of CS B′
Lateral condyle width	LCW	Length from the medial end to the lateral end of CS C′
Medial condyle to middle width	MCMW	Distance between two lines when the lines perpendicular to CS A′ are drawn so as to pass through the ICN and the medial end of CS B′ when observed so as to face CS B′
Lateral condyle to middle width	LCMW	Distance between two lines when lines perpendicular to CS A′ are drawn so as to pass through the ICN and the lateral end of CS C′ when observed so as to face CS C′
Middle-lateral outer angle	MLO-angle	Angle between the straight line that passes through the lateral end of CS C′ at the height of CS A′ and the lateral end of CS C′ at the height of the lateral epicondyle and the straight line perpendicular to CS A′ when observed so as to face CS C′
Front protrusion length	FPL	Distance between two lines when two straight lines parallel to the MA are drawn so as to pass through the point perpendicular to CS D′, which is formed with a cross section that is parallel to SEA and perpendicular to CS A′, which passes through the anterior end of the intercondylar fossa surface of CS A′, from the ICN and the upper end of CS D
Coronal-section outer angle	CSO-angle	Angle formed by the straight line parallel to the MA and the straight-line EF, where point E is the lateral end of CS D′ and point F is the point where the curvature of the outer edge of CS D′ changes from convex to concave
Radiation of the curvature of the lateral anterior excision contour	RLAC	Radius of curvature at the midpoint between point F and the upper end of CS D′
Lateral anterior excision contour width	LAEC	Distance between two lines when straight lines parallel to the MA are drawn on CS D′ so as to pass through point E and the ICN
Medial anterior excision contour width	MAEC	Distance between two lines when straight lines parallel to the MA are drawn on CS D′ so as to pass through the medial end of CS D′ and the ICN
Anterior excision middle-lateral length	AEML	Length of the line segment perpendicular to CS D′ from the front end of the lateral condyle
Anterior excision middle-medial length	AEMM	Length of the line segment perpendicular to CS D′ from the front end of the medial condyle
Patella coronal-section length	PCS	Length of the line segment connecting the point perpendicular to CS D′ from the point at the rear end of the margin connecting the front end of the medial condyle and that of the lateral condyle
Radiation of curvature of the lateral distal condyle	RLDC	Radius of curvature at the point where the straight line connecting the center of the femoral head and the lateral epicondyle intersects the base of the lateral epicondyle when the femur is observed from the lateral side
Radiation of curvature of the lateral posterior condyle	RLPC	Radius of curvature at the midpoint of side GH, where point G, which is orthogonal to CS A′, is the intersection of the edge of the shadow of the lateral condyle projected so as to be perpendicular to plane γ (which is the plane in contact with the lateral epicondyle and orthogonal to CS A′) and CS A′; point H is the intersection of a straight line from point G that is orthogonal to CS A′ and the upper edge of the shadow
Radiation of curvature of the medial distal condyle	RMDC	Radius of curvature at the point where the straight line connecting the center of the femoral head and the medial epicondyle intersects the base of the medial epicondyle when the femur is observed from the medial side
Radiation of curvature of the medial posterior condyle	RMPC	Radius of curvature at the midpoint of side IJ, where point I, which is orthogonal to CS A′, is the intersection of the edge of the shadow of the lateral condyle projected so as to be perpendicular to plane δ (which is the plane in contact with the lateral epicondyle and orthogonal to CS A′) and CS A′; point J is the upper edge of the shadow
Epicondyle axis angle	CEA-angle	Angle formed by a straight line connecting the lateral and medial epicondyles and a straight line passing through the posterior ends of the medial and lateral epicondyles
C-lateral anterior–posterior length	C-LAP	Distance between two lines when lines parallel to the SEA are drawn on the anterior and posterior ends of the lateral condyle on CS A′
C-medial anterior–posterior length	C-MAP	Distance between two lines when lines parallel to SEA are drawn on the anterior and posterior ends of the medial condyle on CS A′

*ICN* intercondylar notch, which is located at the posterior 1/4 point on the midline of the recess between the medial and lateral condyles on the bottom surface of the lower end of the femur, *MA* mechanical axis, which is the axis passing through the center of the femoral head and ICN [29], *SEA* surgical epicondyle axis, which is the axis passing through the medial epicondyle process groove and the lateral epicondyle process [29, 70, 71]

First, to select measurements with acceptable intraobserver and interobserver errors, 20 cadavers were randomly selected. To evaluate the intraobserver error, a single researcher measured the femurs twice with an interval of ≥ 1 day for each cadaver. To evaluate the interobserver error, another researcher measured the femurs, and the result was then compared with the first result from the first researcher. The intraobserver and interobserver errors were assessed with the technical error of measurement (TEM), relative technical error of measurement (rTEM), and coefficient of reliability (R) [49, 50]. The acceptance range for rTEM was set at < 1.5% for intraobserver error and < 2.0% for interobserver error [51].

Second, the sexual differences in age, AS, and acceptable measurement were evaluated. If these values followed a normal distribution, Student’s t-test was used. If the values did not follow a normal distribution, the Wilcoxon rank sum test was used instead [52, 53]. The absolute *z* values of skewness and kurtosis were used to assess normal distribution [54].

Lastly, the relationship between AS and each measurement for all 300 cadavers was assessed using single regression analysis with the statistical values of the coefficient of determination (*R*^2^) and the standard error of the estimate (SEE). In this analysis, all manual markings were performed by a single researcher. A residual plot was created with the predicted stature calculated with the obtained regression equation, and the difference between the predicted stature and AS and the existence of heteroscedasticity was examined [55].

Statistical significance was set at *P* < 0.05 to reject the null hypothesis that there was no significant difference in statistical values between males and females and that the regression coefficient was 0. Statistical analysis was performed using Excel 2010 (Microsoft Corporation, Redmond, WA, USA).

## Results

The 41 measurements were classified into groups 1 and 2 based on the results of intraobserver and interobserver errors (Table 2), and the TEM, rTEM, and *R* values for each measurement of both the right and left femurs are shown in Table 3. Group 1 included measurements with rTEM values < 1.5% for intraobserver error and < 2.0% for interobserver error on both the right and left sides. Group 2 included the other measurements whose rTEM values for intraobserver or interobserver errors were larger than the acceptable range. Group 1 comprised five measurements: maximum length of the femur (MLF), lateral anterior–posterior length (LAP), cross-section medial–lateral width (C-ML), C-lateral anterior–posterior length (C-LAP), and C-medial anterior–posterior length (C-MAP), for which *R* values were > 0.9. Group 2 was classified into groups 2–1 and 2–2 according to measurement type. Group 2–1 included measurements for angles and curvature radii, and group 2–2 included measurements for length.

**Table 2:** Semi-automatic measurement classification

Group		Measurements
1		MLF, LAP, C-ML, C-LAP, C-MAP
2	2–1	VA, LA, PCA-angle, LDA-angle, MDA-angle, JL-angle, MLO-angle, CSO-angle, CEA-angle, MDAC, RLAC, RLDC, RLPC, RMDC, RMPC
	2–2	LRA, MRA, C-P-LAP, C-P-MAP, C-LML, C-MML, P-LAP, P-MAP, MSI, LSI, MCW, LCW, MCMW, LCMW, FPL, LAEC, MAEC, AEML, AEMM, PCS, MAP

Group 1: measurements with rTEM values < 1.5% intraobserver error and < 2.0% interobserver error; group 2: measurements with rTEM values ≥ 1.5% intraobserver error or ≥ 2.0% interobserver error; group 2–1: measurements for angles and radius of curvature; group 2–2: measurements for length.

*MLF* maximum length of the femur, *LAP* lateral anterior–posterior length, *C-ML* cross-section medial–lateral width, *C-LAP* C-lateral anterior–posterior length, *C-MAP* C-medial anterior–posterior length, *MLF* maximum length of the femur, *AEML* anterior excision middle-lateral length, *AEMM* anterior excision middle-medial length, *PCS* patella coronal-section length, *MAEC* medial anterior excision contour width, *RLAC* radiation of the curvature of the lateral anterior excision contour, *LAEC* lateral anterior excision contour width, *RLPC* radiation of curvature of the lateral posterior condyle, *RMPC* radiation of curvature of the medial posterior condyle, *CEA* epicondyle axis angle, *LAP* lateral anterior–posterior length, *C-ML* cross-section medial–lateral width, *C-LAP* C-lateral anterior–posterior length, *C-MAP* C-medial anterior–posterior length.

**Table 3:** TEM, rTEM, and R values for each measurement of both the right and 1 left femurs (n = 20)

	Intraobserver error			Interobserver error		
	TEM (mm)	rTEM (%)	*R*	TEM (mm)	rTEM (%)	*R*
Right MLF	0.14832	0.03506	0.99997	0.07746	0.01831	0.99999
Left MLF	0.14318	0.03374	0.99997	0.08062	0.01900	0.99999
Right VA	0.15572	3.19926	0.98174	0.38923	7.63945	0.89849
Left VA	0.15083	3.13741	0.98725	0.24135	4.90799	0.97020
Right LA	0.31024	10.89524	0.93475	0.56613	23.78686	0.77883
Left LA	0.29917	10.78074	0.92963	0.60704	26.27887	0.77118
Right LAP	0.26173	0.41746	0.99475	0.37550	0.59669	0.98901
Left LAP	0.17103	0.27458	0.99775	0.54658	0.87352	0.97751
Right MAP	0.70054	1.14443	0.96109	1.26323	2.08738	0.86675
Left MAP	0.55159	0.90008	0.97916	1.22913	2.03052	0.89877
Right P-LAP	0.91228	3.84562	0.76938	2.20777	9.97975	0.08897
Left P-LAP	0.96099	4.09629	0.76210	1.99744	9.02898	0.21163
Right P-MAP	1.95371	6.67366	0.45896	5.32863	21.07217	− 0.45641
Left P-MAP	2.09249	7.14159	0.47266	5.63392	22.18077	− 0.51700
Right LRA	0.14832	1.94651	0.98780	0.24495	3.25514	0.96701
Left LRA	0.17321	2.41738	0.97740	0.15572	2.17416	0.98023
Right MRA	0.18841	1.77498	0.97952	0.25348	2.42851	0.96217
Left MRA	0.22694	2.13086	0.97150	0.20797	1.97828	0.97478
Right C-P-LAP	0.98729	5.29168	0.77641	2.29042	13.51676	0.19975
Left C-P-LAP	0.97545	5.34785	0.73812	2.03384	12.06310	0.09859
Right C-P-MAP	2.01041	7.85700	0.38403	5.61095	26.22858	− 0.55040
Left C-P-MAP	2.20528	8.53518	0.49377	5.79055	26.61711	− 0.51468
Right PCA-angle	2.51501	31.90618	0.45966	5.05962	112.74920	− 0.28593
Left PCA-angle	2.35372	28.03717	0.10174	5.40562	114.22340	− 0.48830
Right C-LML	0.79152	2.31743	0.92812	0.98944	2.90373	0.88707
Left C-LML	0.80265	2.31696	0.93975	1.45301	4.29536	0.79284
Right C-MML	0.84202	2.40922	0.90767	1.60008	4.47543	0.69924
Left C-MML	1.18269	3.48080	0.80431	2.49875	7.01748	0.46790
Right C-ML	0.82886	1.14514	0.97573	0.40988	0.56833	0.99405
Left C-ML	0.51745	0.71815	0.99089	0.37683	0.52359	0.99540
Right LDA-angle	2.38065	13.67012	0.78414	4.90607	34.90621	0.26475
Left LDA-angle	2.13395	12.09209	0.81491	5.74861	41.77768	0.16193
Right MDA-angle	2.28561	13.92389	0.79549	4.06789	29.08234	0.33301
Left MDA-angle	2.23523	13.09257	0.76538	5.22705	35.32981	0.10575
Right MDAC	217.05863	202.73064	0.27902	48.28600	73.31891	0.01449
Left MDAC	11.21474	17.03851	0.84829	17.25977	27.19572	0.46401
Right JL-angle	0.20125	4.66928	0.99325	0.24850	5.92714	0.99014
Left JL-angle	0.20797	4.14482	0.98977	0.35249	7.31689	0.96952
Right MSI	1.06243	4.03389	0.83388	2.08273	8.26233	0.55417
Left MSI	0.62510	2.38702	0.93223	2.16038	8.67362	0.53598
Right LSI	0.27839	1.09666	0.99384	0.51210	2.00884	0.98147
Left LSI	0.38633	1.48745	0.98837	0.37081	1.43198	0.98978
Right MCW	1.27112	4.73196	0.75036	2.45260	9.67782	0.44488
Left MCW	0.88204	3.28936	0.86778	2.25832	8.88577	0.61669
Right LCW	0.27523	1.19030	0.99234	0.49975	2.16788	0.97684
Left LCW	0.31024	1.32313	0.99175	0.54521	2.33718	0.97519
Right MCMW	1.02445	2.81211	0.89913	0.82234	2.25748	0.93518
Left MCMW	0.87878	2.44836	0.90267	1.64628	4.49405	0.74669
Right LCMW	0.98362	2.84529	0.88269	1.42241	4.19621	0.79991
Left LCMW	1.00735	2.85833	0.90238	2.16991	6.39525	0.59094
Right MLO-angle	0.78962	3.44737	0.99635	26.35689	143.08842	− 0.72276
Left MLO-angle	1.05345	4.85515	0.99383	26.45265	149.36559	− 0.72506
Right FPL	1.63110	18.80235	0.58494	2.33276	24.43316	0.39590
Left FPL	0.98881	10.58969	0.89538	1.72699	17.73092	0.76812
Right CSO-angle	2.09165	16.92958	0.78946	1.73458	14.6780	0.86539
Left CSO-angle	3.02977	22.55131	0.47633	4.29302	30.80745	0.38151
Right RLAC	1993.09811	133.54248	0.018494	2084.36477	169.53636	− 0.17639
Left RLAC	6665.89030	261.041493	− 0.01652	7335.77418	270.10845	− 0.09082
Right LAEC	0.58758	1.97191	0.94179	0.83830	2.80533	0.89137
Left LAEC	0.84720	2.75894	0.86310	1.10182	3.67456	0.79487
Right MAEC	1.33154	4.95089	0.85018	1.73465	6.69102	0.76575
Left MAEC	1.11018	4.15098	0.78477	1.82941	7.02472	0.56696
Right AEML	2.03568	12.33373	0.67508	3.34066	23.30421	0.27640
Left AEML	1.93035	11.07328	0.54821	3.75955	25.41527	0.11808
Right AEMM	1.11086	11.38172	0.84995	1.40214	15.29053	0.76770
Left AEMM	1.23420	11.35158	0.72772	1.74900	17.74733	0.60414
Right PCS	0.93635	12.43905	0.90597	5.38E + 37	632.45553	− 0.02564
Left PCS	5.38E + 37	632.45553	− 0.02564	5.38E + 37	210.87690	0.63944
Right RLDC	1.00983	3.33194	0.89579	2.17595	7.48971	0.56117
Left RLDC	2.31663	7.59362	0.57532	2.03918	6.99127	0.50181
Right RLPC	0.42574	2.21881	0.97252	0.71028	3.73439	0.93883
Left RLPC	0.46368	2.43722	0.94439	0.95289	4.98765	0.76205
Right RMDC	0.87350	2.44403	0.94240	3.55356	10.64815	0.18188
Left RMDC	1.81859	4.94281	0.87463	4.86654	14.28920	0.08782
Right RMPC	0.33317	1.87172	0.95407	0.51137	2.89646	0.90400
Left RMPC	0.38406	2.18401	0.92483	0.50818	2.90681	0.89752
Right CEA-angle	1.70147	23.45238	0.76024	4.82237	106.63062	− 0.16380
Left CEA-angle	2.46232	31.85402	0.58446	5.92326	139.37081	− 0.16024
Right C-LAP	0.46098	0.97304	0.98342	0.55790	1.17335	0.97425
Left C-LAP	0.41201	0.88819	0.98150	0.74967	1.60048	0.93765
Right C-MAP	0.39686	0.76926	0.98435	0.96203	1.88707	0.90044
Left C-MAP	0.46530	0.90139	0.98600	0.84897	1.66440	0.95143

*TEM* technical error of measurement, *rTEM* relative technical error of measurement, *R* coefficient of reliability, *MLF* maximum length of the femur, *AEML* anterior excision middle-lateral length, *AEMM* anterior excision middle-medial length, *PCS* patella coronal-section length, *MAEC* medial anterior excision contour width, *RLAC* radiation of the curvature of the lateral anterior excision contour, *LAEC* lateral anterior excision contour width, *RLPC* radiation of curvature of the lateral posterior condyle, *RMPC* radiation of curvature of the medial posterior condyle, *CEA* epicondyle axis angle, *LAP* lateral anterior–posterior length, *C-ML* cross-section medial–lateral width, *C-LAP* C-lateral anterior–posterior length, *C-MAP* C-medial anterior–posterior length.

The descriptive statistics for age, AS, and five group 1 measurements are presented in Table 4. Age, AS, MLF, LAP, C-LAP and C-MAP followed a normal distribution, while only C-ML did not follow a normal distribution. There was no significant difference in mean age between the sexes (*P* = 0.482). The mean values of AS and of each measurement were significantly greater in men than in women (C-ML, P < 0.01; AS, MLF, LAP, C-LAP, and C-MAP, *P* < 0.001).

**Table 4:** Descriptive statistics for age, AS, and group 1 measurements

	All cadavers (*n* = 300)		Male (*n* = 150)		Female (*n* = 150)		*F* value	*P* value
	Range	Mean ± SD	Range	Mean ± SD	Range	Mean ± SD		
Age (years)	18–88	50.35 ± 17.84	19–84	49.62 ± 15.15	18–88	51.07 ± 20.21	0.4967	0.482
AS (cm)	141–184	162.2 ± 9.84	152–184	169.6 ± 6.73	141–177	154.8 ± 6.15	398.5	< 0.001
MLF (cm)								
Right	36.14 –50.95	42.69 ± 2.920	39.07–50.95	44.71 ± 2.118	36.14–48.65	40.66 ± 2.092	27.72	< 0.001
Left	35.83–51.27	42.79 ± 2.960	38.95–51.27	44.86 ± 2.106	35.83–48.95	40.71 ± 2.116	28.98	< 0.001
Average	36.06–51.11	42.74 ± 2.937	39.01–51.11	44.79 ± 2.108	36.06–48.80	40.69 ± 2.099	28.47	< 0.001
LAP (cm)								
Right	5.16–7.47	6.30 ± 0.470	5.86–7.47	6.638 ± 0.353	5.16–6.89	5.962 ± 0.298	32.12	< 0.001
Left	5.26–7.46	6.289 ± 0.463	5.84–7.46	6.627 ± 0.338	5.26–6.78	5.951 ± 0.292	34.30	< 0.001
Average	5.235–7.43	6.295 ± 0.464	5.88–7.43	6.633 ± 0.342	5.24–6.84	5.957 ± 0.292	33.98	< 0.001
C-ML (cm)								
Right	5.92–8.66	7.278 ± 0.628	6.97–8.66	7.808 ± 0.366	5.92–7.72	6.748 ± 0.303	-	< 0.01
Left	5.95–8.71	7.243 ± 0.629	6.81–8.71	7.776 ± 0.365	5.95–7.59	6.710 ± 0.297	-	< 0.01
Average	5.94–8.58	7.261 ± 0.629	6.90–8.58	7.792 ± 0.361	5.94–7.62	6.729 ± 0.292	-	< 0.01
C-LAP (cm)								
Right	3.47–5.84	4.686 ± 0.426	4.25–5.84	4.941 ± 0.357	3.47–5.09	4.431 ± 0.325	16.75	< 0.001
Left	3.75–5.72	4.667 ± 0.398	4.00–5.72	4.889 ± 0.351	3.75–5.14	4.446 ± 0.308	13.51	< 0.001
Average	3.63–5.68	4.677 ± 0.401	4.23–5.68	4.915 ± 0.343	3.63–5.10	4.438 ± 0.301	16.42	< 0.001
C-MAP (cm)								
Right	4.33–6.27	5.258 ± 0.371	4.58–6.27	5.483 ± 0.307	4.33–5.76	5.033 ± 0.283	17.43	< 0.001
Left	4.27–6.41	5.240 ± 0.377	4.69–6.41	5.484 ± 0.302	4.27–5.71	4.997 ± 0.272	21.56	< 0.001
Average	4.30–6.34	5.249 ± 0.367	4.71–6.34	5.435 ± 0.296	4.30–5.74	5.019 ± 0.270	20.56	< 0.001

Wilcoxon rank sum test was used to estimate the *P* value in the C-ML values, and Student’s *t*-test was used to estimate the P value in the other measurements. *AS* adjusted stature, which was calculated by subtracting 2.0 cm from the measured stature; *SD* standard deviation; *AZS* absolute *Z* value of skewness; *AZK* absolute *Z* value of kurtosis; MLF, maximum length of the femur; *LAP* lateral anterior–posterior length; *C-ML* cross-section medial–lateral width; C*-LAP* C-lateral anterior–posterior length; *C-MAP* C-medial anterior–posterior length.

**Fig. 3:**
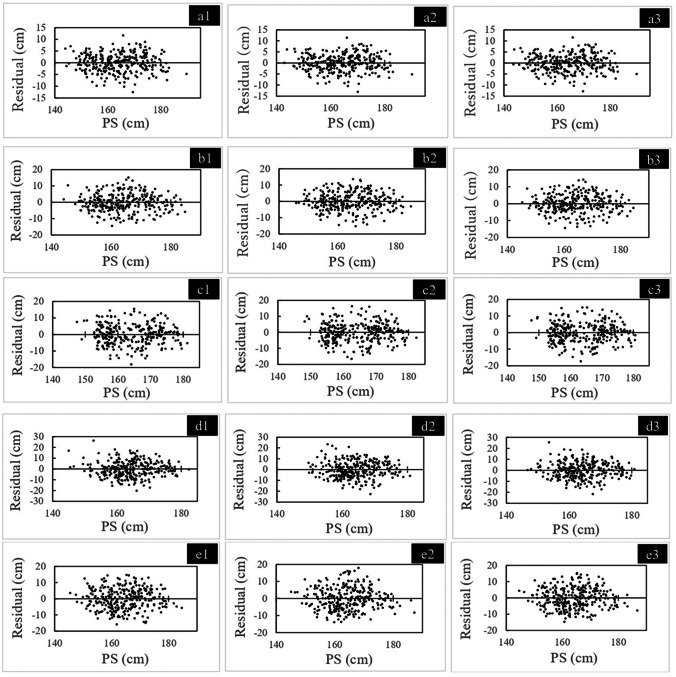
Residual distribution for all samples regardless of sex with the five measurements. **a1**: Right MLF (maximum length of the femur): **a2**: left MLF, a3: average MLF; **b1**: right LAP (lateral anterior–posterior length): **b2**: left LAP, **b3**: average LAP; **c1**: right C-ML (cross-section medial–lateral width): **c2**: left C-ML, **c3**: average C-ML; **d1**: right C-LAP (C-lateral anterior–posterior length): **d2**: left C-LAP, **d3**: average C-LAP; **e1**: right C-MAP (C-medial anterior–posterior length), **e2**: Left C-MAP, **e3**: average C-MAP. AS, adjusted stature, PS, predicted stature calculated with the obtained regression equation

**Fig. 4:**
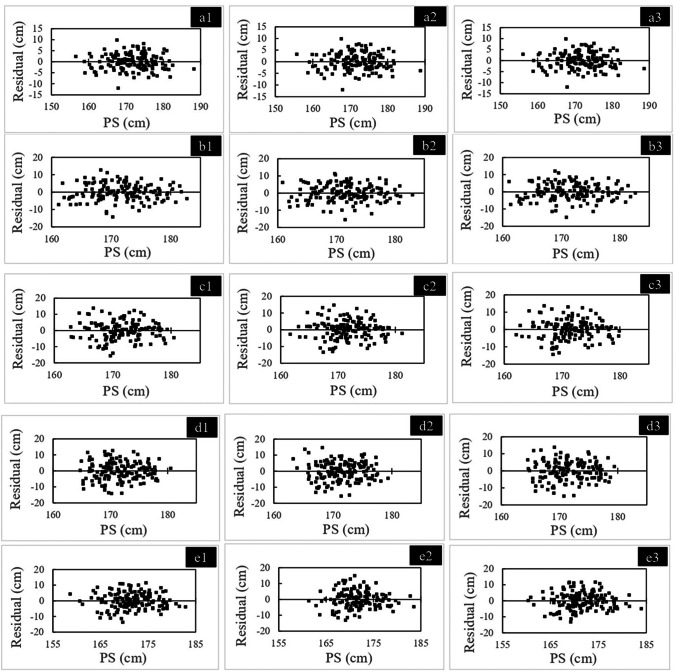
Residual distribution for male samples with the five measurements. **a1**: right MLF (maximum length of the femur): **a2**: left MLF, **a3**: average MLF; **b1**: right LAP (lateral anterior–posterior length): **b2**: left LAP, b3: average LAP; **c1**: right C-ML (cross-section medial–lateral width): **c2**: left C-ML, **c3**: Average C-ML; **d1**: right C-LAP (C-lateral anterior–posterior length): **d2**: left C-LAP, d3: average C-LAP; **e1**: right C-MAP (C-medial anterior–posterior length): **e2**: left C-MAP, e3: average C-MAP. AS, adjusted stature, PS: predicted stature calculated with the obtained regression equation

**Fig. 5:**
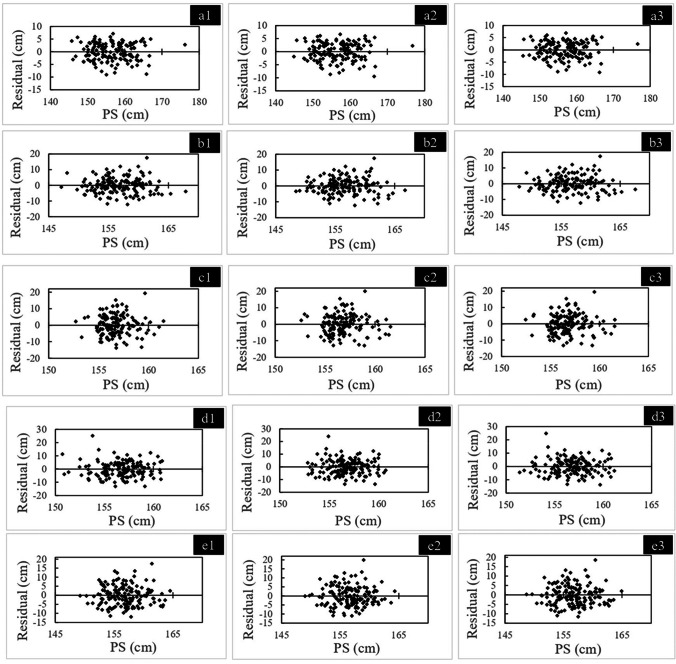
Residual distribution for female samples with the five measurements. **a1**: right MLF (maximum length of the femur): **a2**: left MLF, **a3**: average MLF; **b1**: right LAP (lateral anterior–posterior length): **b2**: left LAP, **b3**: average LAP; **c1**: right C-ML (cross-section medial–lateral width): **c2**: left C-ML, **c3**: average C-ML; d1: right C-LAP (C-lateral anterior–posterior length): d2: left C-LAP, d3: average C-LAP; e1: right C-MAP (C-medial anterior–posterior length): e2: left C-MAP, e3: average C-MAP. AS, adjusted stature; PS, predicted stature calculated with the obtained regression equation

Table 5 describes the result of the single linear regression analysis for estimating AS using five group 1 measurements for all cadavers, regardless of sex. Tables 6 and 7 show the results for males and females, respectively. Significant positive correlations were observed between the AS and each measurement. MLF had the strongest correlation and the lowest SEE for all cadavers, while LAP had the second strongest correlation and lowest SEE. Figures 3, 4, and 5 show the residual plots for the five measurements.

**Table 5:** Simple linear regression analyses for stature estimation for all samples regardless of sex

	Side	Regulation formula (cm)	SEE (cm)	*R*^2^	*P* value
MLF (cm)	Right	*y* = 3.091*x* + 32.230	3.913	0.842	< 0.001
	Left	*y* = 3.060 *x* + 33.261	3.837	0.848	< 0.001
	Average	*y* = 3.083 *x* + 32.442	3.850	0.847	< 0.001
LAP (cm)	Right	*y* = 17.582 *x* + 53.413	5.340	0.706	< 0.001
	Left	*y* = 17.813 *x* + 52.159	5.340	0.702	< 0.001
	Average	*y* = 17.896 *x* + 51.533	5.287	0.712	< 0.001
C-ML (cm)	Right	*y* = 12.358 *x* + 74.240	6.047	0.623	< 0.001
	Left	*y* = 12.380 *x* + 74.518	6.019	0.627	< 0.001
	Average	*y* = 12.488 *x* + 73.514	5.985	0.631	< 0.001
C-LAP (cm)	Right	*y* = 15.768 *x* + 90.296	7.199	0.466	< 0.001
	Left	*y* = 15.786 *x* + 90.507	7.583	0.408	< 0.001
	Average	*y* = 16.685 *x* + 86.153	7.226	0.462	< 0.001
C-MAP (cm)	Right	*y* = 20.192 *x* + 58.015	6.384	0.580	< 0.001
	Left	*y* = 19.710 *x* + 60.897	6.461	0.570	< 0.001
	Average	*y* = 20.656 *x* + 55.757	6.267	0.595	< 0.001

*SEE* standard error of the estimate, *MLF* maximum length of the femur, *LAP* lateral anterior–posterior length, *C-ML* cross-section medial–lateral width, *C-LAP* C-lateral anterior–posterior length, *C-MAP* C-medial anterior–posterior length.

**Table 6:** Simple linear regression analyses for stature estimation in males

	Side	Regulation formula (cm)	SEE (cm)	*R*^2^	*P* value
MLF (cm)	Right	*y* = 2.667*x* + 52.355	3.664	0.705	< 0.001
	Left	*y* = 2.682*x* + 51.321	3.667	0.705	< 0.001
	Average	*y* = 2.686*x* + 51.298	3.646	0.708	< 0.001
LAP (cm)	Right	*y* = 13.391*x* + 82.717	4.807	0.493	< 0.001
	Left	*y* = 13.688*x* + 80.906	4.897	0.474	< 0.001
	Average	*y* = 13.860*x* + 79.686	4.797	0.495	< 0.001
C-ML (cm)	Right	*y* = 10.467*x* + 89.882	5.547	0.325	< 0.001
	Left	*y* = 10.266*x* + 91.779	5.608	0.310	< 0.001
	Average	*y* = 10.609*x* + 88.945	5.547	0.325	< 0.001
C-LAP (cm)	Right	*y* = 9.921*x* + 122.590	5.739	0.277	< 0.001
	Left	*y* = 9.348*x* + 125.910	5.891	0.238	< 0.001
	Average	*y* = 10.305*x* + 120.960	5.747	0.275	< 0.001
C-MAP (cm)	Right	*y* = 14.347*x* + 92.950	5.106	0.428	< 0.001
	Left	*y* = 12.911*x* + 100.810	5.504	0.335	< 0.001
	Average	*y* = 14.418*x* + 92.554	5.220	0.402	< 0.001

*SEE* standard error of the estimate, *MLF* maximum length of the femur, *LAP* lateral anterior–posterior length, *C-ML* cross-section medial–lateral width, *C-LAP* C-lateral anterior–posterior length, *C-MAP* C-medial anterior–posterior length.

**Table 7:** Simple linear regression analyses for stature estimation in females

	Side	Regulation formula (cm)	SEE (cm)	*R*^2^	*P* value
MLF (cm)	Right	*y* = 2.434 *x* + 57.802	3.456	0.686	< 0.001
	Left	*y* = 2.428 *x* + 57.895	3.384	0.699	< 0.001
	Average	*y* = 2.442 *x* + 57.409	3.403	0.696	< 0.001
LAP (cm)	Right	*y* = 12.007 *x* + 85.171	5.012	0.340	< 0.001
	Left	*y* = 12.044 *x* + 85.083	5.060	0.327	< 0.001
	Average	*y* = 12.319 *x* + 83.375	5.005	0.341	< 0.001
C-ML (cm)	Right	*y* = 4.877 *x* + 123.850	5.986	0.058	0.003
	Left	*y* = 5.440 *x* + 120.260	5.951	0.069	0.001
	Average	*y* = 5.424 *x* + 120.260	5.959	0.067	0.001
C-LAP (cm)	Right	*y* = 6.287 *x* + 128.900	5.817	0.110	< 0.001
	Left	*y* = 5.662 *x* + 131.590	5.914	0.081	< 0.001
	Average	*y* = 6.647 *x* + 127.260	5.833	0.106	< 0.001
C-MAP (cm)	Right	*y* = 10.698 *x* + 102.920	5.367	0.243	< 0.001
	Left	*y* = 10.626 *x* + 103.670	5.441	0.222	< 0.001
	Average	*y* = 11.317 *x* + 100.000	5.354	0.247	< 0.001

*SEE* standard error of the estimate, *MLF* maximum length of the femur, *LAP* lateral anterior–posterior length, *C-ML* cross-section medial–lateral width, *C-LAP* C-lateral anterior–posterior length, *C-MAP* C-medial anterior–posterior length.

## Discussion

In this study, we obtained stature estimation formulae based on a 3D model reconstructed from CT images using semi-automatic measurement software. This is the first report that obtained stature estimation formulae from measurements in 3D CT-reconstructed images using semi-automatic measurement software. In the present study, artificial intelligence (AI) was used for recognition of the femur, extraction of bone surface information, and semi-automatic measurement. AI has been applied in multiple fields of medical research. In the field of diagnostic imaging, it has been shown to reduce not only time for analysis but also interreader variability or false-positive markings [56–59]. Furthermore, AI has been shown to improve adenoma detection rates and reduce examination time in colonoscopy [60], thus reducing waiting time for outpatients [61] and the time interval between CT angiography at a primary stroke center to door-in at a comprehensive stroke center [62]. In the present study, the advantages of using a semi-automatic measurement software were the following: it is a simple measurement method; the time required for measurement is short (approximately 1 min); multiple measurements can be obtained with a single method.

Previously, some stature estimation methods with a single linear regression analysis from MLF measured using radiographic images were reported (Table 8). In two previous reports that presented intraobserver and interobserver errors [40, 44], the rTEM values for intraobserver errors were 0.108–0.277 and those for interobserver errors were 0.192–0.289. In this report, the rTEM values for intraobserver errors were 0.034–0.035 and those for interobserver errors were 0.018–0.019, which were lower than in these two reports. It is possible that these errors were reduced using semi-automatic measurement software.

**Table 8:** Outline of previous and present studies having performed simple linear regression analysis for stature estimation with maximum length of the femur whose length was measured using radiographic images

Author	Subject	Equipment	Side	Sex	Stature estimation formula	Correlation coefficient	*R*^2^	SEE (cm)	Intra-OE (%)	Inter-OE (%)
Present study	Japanese	Computed tomography	Right	Male (*n* = 150)	*y* = 2.667*x* + 52.355	-	0.7053	3.664	0.035	0.018
				Female (*n* = 150)	*y* = 2.682 *x* + 51.321	-	0.7049	3.667		
			Left	Male (*n* = 150)	*y* = 2.434 *x* + 57.802	-	0.686	3.456	0.034	0.019
				Female (*n* = 150)	*y* = 2.428 *x* + 57.895	-	0.6989	3.384		
Chiba et al. [44]	Japanese	Computed tomography	Right	Male (*n* = 116)	*y* = 2.670 *x* + 50.987	-	0.654	3.847	0.108	0.205
				Female (*n* = 108)	*y* = 2.702 *x* + 46.624	-	0.768	3.29		
			Left	Male (*n* = 116)	*y* = 2.748 *x* + 47.259	-	0.653	3.85	0.247	0.289
				Female (*n* = 108)	*y* = 2.646 *x* + 48.733	-	0.76	3.34		
Hasegawa et al. [37]	Japanese	X-ray photography	Right	Male (*n* = 92)	*y* = 2.42 *x* + 63.32	0.895	0.8010*	3.77	-	-
				Female (*n* = 342)	*y* = 2.31 *x* + 62.05	0.809	0.6545*	3.03		
			Left	Male (*n* = 92)	*y* = 2.49 *x* + 60.30	0.903	0.8154*	3.83	-	-
				Female (*n* = 342)	*y* = 2.28 *x* + 63.37	0.813	0.6610*	3.00		
Hishmat et al. [40]	Japanese	Computed tomography	Right	Male (*n* = 150)	*y* = 2.61 *x* + 52.17	0.73	0.5329*	4.83	0.149	0.192
				Female (*n* = 109)	*y* = 2.86 *x* + 38.48	0.83	0.6889*	4.29		
			Left	Male (*n* = 150)	*y* = 2.47 *x* + 58.04	0.72	0.5184	4.72	0.277	0.201
				Female (*n* = 109)	*y* = 2.87 *x* + 37.42	0.81	0.6561*	4.49		
Nishio [42]	Japanese	Computed tomography	Right	Male (*n* = 215)	*y* = 3.20 *x* + 27.81	0.8967	0.8041*	4.32	-	-
				Female (*n* = 120)	*y* = 3.36 *x* + 20.22	0.8784	0.7716*	4.12		
			Left	Male (*n* = 215)	*y* = 3.11 *x* + 31.13	0.8841	0.7816*	4.60	-	-
				Female (*n* = 120)	*y* = 3.15 *x* + 29.00	0.8591	0.7381*	4.39		
Zhang et al. [63]	Danish	Computed tomography	Right	Male (*n* = 41)	*y* = 2.23 *x* + 70.70	-	0.52	4.4	-	-
				Female (*n* = 37)	*y* = 2.13 *x* + 69.26	-	0.75	3.5		
			Left	Male (*n* = 41)	*y* = 2.19 *x* + 72.17	-	0.51	4.4	-	-
				Female (*n* = 37)	*y* = 2.07 *x* + 72.03	-	0.74	3.6		
Lee et al. [39]	Korean	Computed tomography	Right	Male (*n* = 155)	*y* = 2.593 *x* + 54.081	0.85	0.722*	3.305	-	-
				Female (*n* = 153)	*y* = 2.82 *x* + 41.926	0.89	0.792*	3.417		
			Left	Male (*n* = 155)	*y* = 2.610 *x* + 54.081	0.859	0.737*	3.214	-	-
				Female (*n* = 153)	*y* = 2.842 *x* + 40.776	0.886	0.785*	3.468		

^*^The values in the *R*^2^ segments are not presented in the reports but were calculated by the square of the correlation coefficient presented with them for ease of comparison.

*SEE* standard error of the estimate, *intra-OE and Inter-OE* r-TEMs of intraobserver and interobserver errors, respectively.

Compared with previous reports [37, 40, 42, 44] of Japanese cadavers, the results of R^2^ and SEE in this study were either better or at least not inferior; therefore, the stature estimation formulae determined in this study could be useful in forensic medical practice. Compared with previous reports providing stature estimations using CT images of Japanese femurs [40, 42, 44], the present study observed the lowest SEE in males, whereas the SEE in females was the second lowest after Chiba et al. [44], and the difference was < 0.2 cm. In their report, MLF was manually measured by reproducing the conventional anthropological measurement method using a CT arbitrary cross-section reconstruction image. Although it may be highly applicable to conventional bone measurements, their measurement method is complicated and time consuming, taking approximately 140 s for measuring MLF, and approximately 440 s for measuring the 5 measurements needed for single side written in the research [44]. In contrast, the semi-automatic measurement method examined in this study is much simpler and faster. It took approximately 40 s from manual marking to displaying 41 measurements, and approximately 280 s from launching this application to displaying all the results. This time period includes measurements of both sides of the femur and includes the time required for 3D model reconstruction. Since the semi-automatic measurement method reduced the measurement error and shortened the measurement time, it is expected that if a fully automatic measuring method is developed, it will be possible to measure with smaller errors and shorter measurement time than the results of this study currently show.

Hasegawa et al. [37] showed lower SEE values in females than those observed in this study (difference, > 0.3 cm), and their report showed the best results in terms of SEE in Japanese subjects, as shown in Table 8 [37, 40, 42, 44]. However, the SEE in males was slightly higher than that observed in males in this study. In addition, the difference in SEE between males and females was 0.74 and 0.83, which was greater than difference in this study (0.003 and 0.072). Hasegawa et al. [37] provided stature estimation formulae using an X-ray photograph of a living human. The difference between this report and theirs might be because their patients were alive, the radiation imaging device was different, and the number of female samples was higher than that of the male samples in their study.

Comparison of the present study with those of Zhang et al. [63] and Lee et al. [39] is complicated because the subjects are different, but our results were superior to those of Zhang et al. [63] and slightly inferior to those of Lee et al. [39]. Zhang et al. [63] studied a smaller number of cadavers than this study; therefore, the difference might be due to the sample size. Meanwhile, Lee et al. [39] had more cadavers with age of 41–60 years (65.8% for men and 45.1% for women) than our report (50.0% for men and 30.0% for women). They might have obtained better results of stature estimation formulae than this study, whose age groups of cadavers were scattered because their stature estimation formulae were adapted to the age groups that comprised most of their cadavers. The difference in age composition ratio, CT equipment, or image reconstruction software may have affected the results.

Many reports have shown that MLF is useful for stature estimation, consistent with our finding that stature estimation with MLF showed the best performance. However, it is impossible to measure MLF if only part of the femur remains. In this study, stature estimation using LAP showed the second lowest SEE. This suggests that LAP would be useful for stature estimation if the MLF cannot be measured, for example, if only the lower part of the femur remains. Although some reports provided stature estimation formulae using measurements of the lower part of the femur [29, 44, 64, 65], no report has suggested that LAP is useful for stature estimation. The high values of SEE for LAP and the three measurements, C-ML, C-LAP, and C-MAP, were not negligible. However, of all the studies that performed stature estimation using the measurements of the lateral side of the femur, only Chiba et al. [44] calculated SEE. They reported that the SEEs calculated from femoral epicondylar breadth (linear distance between projection points of the most medial and lateral epicondyles projected vertically to the horizontal) was 5.620–6.300. Compared to their study, SEEs calculated from LAP showed better results, and SEEs calculated from other measurements were not inferior. Since there are few comparison targets, further research on stature estimation using the measurements of the distal part of the femur is desirable in future studies.

Among the 41 measurements that were semi-automatically measured in the present study, group 2 measurements had large intraobserver and interobserver errors outside the permissible range. Descriptive statistics for the measurements corresponding to group 2 are shown in Online Resource 1. There are several possible reasons for the higher measurement errors in group 2 measurements. Group 2–1 measurements were based on information from the edge of the reconstructed 3D CT model. Therefore, the slight difference in construction due to the manual removal of calcified blood vessels and cartilage might have resulted in a large error. Group 2–2 measurements, except MAP, had smaller values than those of group 1, as shown in Table 4 and Online Resource 1. Therefore, the error caused by manual operation might have had a significant influence on these measurements. MAP had similar values to those of group 1 measurements, but it also had higher measurement errors. Unlike C-ML, C-LAP, and C-MAP, MAP is measured without creating a cross section at the lower part of the femur. The deformation of the knee joint, including the distal end of the femur, might have occurred in most of the cadavers in this study because primary knee osteoarthritis often occurs in people over 50 years old [66, 67]. This change may have made it difficult for the AI software to have identified them. In some cadavers, the software used in this study mistakenly recognized the knee cartilage and patella as part of the femur when it identified the femur, and the structure other than the femur had to be manually removed. This manual operation might have caused higher measurement errors. In addition, MAP had a larger measurement error than LAP, which was also measured without creating a cross section. This may be because osteoarthritis occurs more frequently on the medial side than on the lateral side [64].

The residual plots indicated that the two measurements, MLF and LAP, were good models for calculating regression equations. The other three measurements were difficult to adopt for the regression equations, because of the large outliers and a small range of predicted values, especially in the residual plots using single-sex. This may be attributed to the small range of the three measurements.

This study has several limitations. The measurements useful in other reports, such as the femoral diaphysis length, physiological length, or bicondylar length [13, 29, 65, 68, 69], were not measured because the semi-automatic measurement application was not configured to measure them. Furthermore, the application was developed by Fujifilm, including measurements selection. The femurs measured in this study were collected only from cadavers with soft tissue, so further studies examining the difference between digital and analog measurements are warranted. Femur deformation due to aging was not considered. In this research, the stature of the cadavers was recalculated in AS, and the estimation formulae were assessed, but since the actual stature was measured only once, intra- and inter-observer errors were not evaluated for the stature. Age-stratified analysis was not performed because of the insufficient sample size in this study. In addition, this study was performed using images captured with two types of CT equipment. Further studies comparing and examining images acquired with different CT devices are warranted.

## Conclusion

This study provided the first stature estimation formulae based on a 3D CT model of modern Japanese femurs using a simple and rapid semi-automatic measurement software. For stature estimation with this method, MLF was the best, and LAP was the second-best measurement using 41 total measurements. These formulae can be useful in forensic investigations.

## Supplementary Information

Below is the link to the electronic supplementary material.

Supplementary file1 (PDF 212 KB)

## Data Availability

The datasets generated and analyzed during the current study are available from the corresponding author on reasonable request.

## Code availability

Code sharing not applicable to this article as no codes were generated or analyzed during the current study.
