# Supplementary material for: Stature estimation by semi-automatic measurements of 3D CT images of the femur
Source: Int J Legal Med. 2022 Dec 7;137(2):359–77. doi: 10.1007/s00414-022-02921-y (PMC9902306; doi:10.1007/s00414-022-02921-y)
Supplement: Supplementary file 1 — Supplementary file1 (PDF 212 KB) [file 414_2022_2921_MOESM1_ESM.pdf]

Title: Stature estimation by semi-automatic measurements of 3D CT images of the femur

Journal: *International Journal of Legal Medicine*

Kei Kira[1,2], Fumiko Chiba[1,2], Yohsuke Makino[1,2], Suguru Torimitsu[1,2], Rutsuko Yamaguchi[1,2], Shigeki Tsuneya[1,2], Ayumi Motomura[1, 2, 3], Maiko Yoshida[2], Naoki Saitoh[2], Go Inokuchi[1, 2], Yumi Hoshioka[2], Hisako Saitoh[2], Daisuke Yajima[2, 3], Hirotaro Iwase[1,2]

[1]Department of Forensic Medicine, Graduate School of Medicine, The University of Tokyo, 7-3-1 Hongo, Bunkyo-ku, Tokyo, Japan

[2]Department of Legal Medicine, Graduate School of Medicine, Chiba University, 1-8-1 Inohana, Chuo-ku, Chiba city, Chiba, Japan

[3]Department of Forensic Medicine, School of Medicine, International University of Health and Welfare, 4-3 Kozunomori, Narita, Chiba, Japan

Corresponding author: Kei Kira

Department of Forensic Medicine, Graduate School of Medicine, The University of Tokyo, 7-3-1 Hongo, Bunkyo-ku, Tokyo, Japan

Department of Legal Medicine, Graduate School of Medicine, Chiba University, 1-8-1 Inohana, Chuo-ku, Chiba city, Chiba, Japan

E-mail: kei10minaho0614@gmail.com

Online Resource 1: Descriptive statistics for measurements of group 2 (excluded outliers in PCS; male: n = 149, female: n = 148)

	All subjects (n = 300)		Male (n = 150)		Female (n = 150)		F value	P value
	Range	Mean ± SD	Range	Mean ± SD	Range	Mean ± SD		
VA (°)								
Right	1.5–10.1	4.476 ± 1.191	1.5–7	4.455 ± 0.976	1.7–10.1	4.497 ± 1.380	0.092	0.761
Left	1.9–10.1	4.662 ± 1.159	1.9–7.6	4.589 ± 0.932	2.1–10.1	4.735 ± 1.348	1.186	0.277
Average	2.05–9.8	4.569 ± 1.127	2.1–7.2	4.522 ± 0.900	2.05–9.8	4.616 ± 1.317	6.519	0.472
LA (°)								
Right	–0.2–7.1	3.207 ± 1.275	1–7.1	3.416 ± 1.193	–0.2–7	2.298 ± 1.323	8.228	0.004
Left	0.3–7.5	3.221 ± 1.311	0.6–6.8	3.426 ± 1.187	0.3–7.5	3.016 ± 1.399	7.465	0.007
Average	0.05–7.25	3.214 ± 1.215	1.1–6.3	3.421 ± 1.110	0.05–7.25	3.007 ± 1.283	8.903	0.003
PCA-angle (°)								
Right	–2.2–16.5	8.592 ± 2.679	–1.5–12.8	8.153 ± 2.680	–2.2–16.5	9.031 ± 2.614	8.221	0.004
Left	–3.2–17.4	8.967 ± 2.778	–3.1–16.3	8.633 ± 2.589	–3.2–17.4	9.301 ± 2.614	4.371	0.374
Average	1.55–14.1	8.780 ± 2.253	1.55–12.7	8.393 ± 2.193	1.85–14.1	9.166 ± 2.253	9.034	0.003
LDA-angle (°)								
Right	–6.4–29.2	17.62 ± 4.965	5.8–28.4	18.50 ± 4.214	–6.4–29.2	16.75 ± 5.492	9.562	0.002
Left	1.9–32.4	17.36 ± 4.927	3.9–28.3	18.63 ± 4.632	1.5–32.4	16.10 ± 4.992	20.68	<0.001
Average	1.55–30.35	17.49 ± 4.493	8.8–28.35	18.56 ± 3.935	1.55–30.35	16.42 ± 4.752	17.95	<0.001
MDA-angle (°)								
Right	4.8–2.94	16.63 ± 0.411	7.6–29.4	16.81 ± 4.230	4.8–27.5	16.45 ± 3.993	0.575	0.449
Left	2.2–27.7	16.34 ± 3.970	7–27.7	16.71 ± 3.917	2.2–24.2	15.98 ± 4.002	2.540	0.112

Average	5.8–28.55	16.48 ± 3.664	8.2–28.55	16.76 ± 3.638	5.8–24.05	16.21 ± 3.682	1.659	0.199
JL-angle (°)								
Right	−1.5–13.7	5.572 ± 2.488	−1.5–10.8	4.818 ± 2.010	−1.2–13.7	6.326 ± 2.690	30.14	<0.001
Left	−2.8–12.3	5.530 ± 2.317	−2.1–10.1	5.004 ± 1.794	−2.8–12.3	6.057 ± 2.645	16.22	<0.001
Average	−1.8–12.25	5.551 ± 2.228	−1.8–10.2	4.911 ± 1.758	−1.1–12.25	6.191 ± 2.458	26.38	<0.001
MLO-angle (°)								
Right	−3.7–76.7	19.66 ± 8.709	3–29.8	18.27 ± 4.627	−3.7–76.7	21.06 ± 11.265	7.828	0.005
Left	−90–76.8	18.72 ± 11.430	1.6–30.8	18.14 ± 4.748	−90–76.8	19.30 ± 15.458	0.759	0.384
Average	−32.75–76.35	19.19 ± 8.288	6–29.5	18.21 ± 4.182	−32.75–76.35	20.18 ± 10.882	4.266	0.040
CSO-angle (°)								
Right	0.9–25.3	12.22 ± 4.022	0.9–25.3	12.21 ± 4.243	2.7–20.8	12.23 ± 3.804	0.001	0.974
Left	0.9–26.1	13.14 ± 4.196	2.5–23.8	12.95 ± 4.352	1.1–26.1	13.33 ± 4.041	0.597	0.440
Average	2–22.85	12.68 ± 3.462	3.25–22.85	12.58 ± 3.639	2–20.65	12.78 ± 3.285	0.237	0.626
CEA-angle (°)								
Right	−10.3–20.5	7.662 ± 3.521	0–20.5	7.51 ± 3.362	−10.3–17.9	7.815 ± 3.362	0.559	0.455
Left	−11.6–22.6	7.694 ± 4.103	−2.5–21.4	7.91 ± 3.710	−11.6–22.6	7.481 ± 4.465	0.808	0.370
Average	−10.95–19.1	7.678 ± 3.385	0.15–19.1	7.71 ± 3.053	−10.95–18	7.648 ± 3.698	0.024	0.867
MDCA (cm)								
Right	2.33–167.12	8.44 ± 15.620	2.95–84	8.84 ± 11.749	2.33–167.12	8.04 ± 187.406	0.200	0.655
Left	2.14–43,437.87	153.38 ± 2507.432	2.62–149.07	9.85 ± 16.114	2.14–43,437.87	29,691 ± 35,461.09	0.980	0.323
Average	24.65–217,350.8	80.91 ± 1254.436	2.80–81.73	9.35 ± 10.809	2.47–21,735.08	152.48 ± 1774.08	0.973	0.325
RLAC (cm)								

Right	1.29–119,726.9	835.97 ± 8218.692	3.66–119,726.9	1151.15 ± 115,95.89	1.29–1744.46	120.79 ± 2292.239	2.274	0.133
Left	1.41–941,313.1	3494.22 ± 54,365.79	1.76–941,313.1	6660.73 ± 76,860.18	1.41–21,360.29	327.70 ± 18,546.92	1.014	0.315
Average	1.51–470,844.3	2165.09 ± 27,478.41	6.06–470,844.3	4105.94 ± 38,816.41	1.51–10,740.43	224.25 ± 946.45	1.494	0.223
RLDC (cm)								
Right	1.84–6.31	3.13 ± 0.444	2.38–4.13	3.16 ± 0.347	1.84–6.31	3.09 ± 5.215	1.954	0.163
Left	2.02–4.86	3.09 ± 0.383	2.43–4.86	3.15 ± 0.351	2.02–4.27	3.03 ± 4.040	7.802	0.006
Average	1.47–2.49	3.11 ± 0.350	2.41–3.77	3.16 ± 0.300	2.09–4.95	3.06 ± 0.389	5.821	0.016
RLPC (cm)								
Right	1.42–2.61	1.92 ± 0.221	1.68–2.61	2.04 ± 0.176	1.42–2.61	1.79 ± 1.614	170.32	<0.001
Left	1.38–2.63	1.92 ± 0.204	1.61–2.63	2.03 ± 0.182	1.38–2.21	1.80 ± 1.522	141.81	<0.001
Average	1.47–2.49	1.92 ± 0.198	1.67–2.49	2.04 ± 0.168	1.47–2.39	1.80 ± 0.144	180.99	<0.001
RMDC (cm)								
Right	2.35–4.8	3.61 ± 0.421	2.98–4.61	3.81 ± 0.350	2.35–4.8	3.42 ± 3.936	82.78	<0.001
Left	2.6–5.44	3.63 ± 0.450	3.08–5.44	3.85 ± 0.415	2.6–4.81	3.41 ± 3.670	97.43	<0.001
Average	2.67–4.88	3.62 ± 0.393	3.16–4.88	3.83 ± 0.347	2.67–4.60	3.41 ± 0.318	118.92	<0.001
RMPC (cm)								
Right	1.45–3.09	1.85 ± 0.184	1.56–2.32	1.93 ± 0.162	1.45–3.09	1.76 ± 0.162	87.29	<0.001
Left	1.44–2.57	1.84 ± 0.178	1.56–2.46	1.93 ± 0.162	1.44–2.57	1.75 ± 1.443	102.57	<0.001
Average	1.51–2.71	1.84 ± 0.173	1.59–2.39	1.93 ± 0.156	1.51–2.71	1.75 ± 0.141	106.27	<0.001
MAP (cm)								
Right	5.23–7.37	6.23 ± 0.442	5.75–7.37	6.53 ± 0.342	5.23–6.74	5.94 ± 0.318	236.1	<0.001
Left	5.21–7.47	6.24 ± 0.453	5.82–7.47	6.55 ± 0.348	5.21–6.77	5.92 ± 0.312	263.9	<0.001
Average	5.24–7.42	6.24 ± 0.443	5.79–7.42	6.54 ± 0.340	5.24–6.76	5.93 ± 0.309	284.7	<0.001

P-LAP (cm)									
Right	1.63–3.11	2.39 ± 0.249	1.98–3.11	2.54 ± 0.208	1.63–2.83	2.24 ± 0.186	177.5	<0.001
Left	1.83–3.07	2.37 ± 0.251	1.94–3.41	2.50 ± 0.219	1.83–2.78	2.21 ± 0.186	158.3	<0.001
Average	1.83–3.07	2.37 ± 0.231	1.96–3.07	2.52 ± 0.186	1.83–2.81	2.22 ± 0.167	216.3	<0.001
P-MAP (cm)								
Right	1.66–3.92	2.99 ± 0.276	2.34–3.92	3.16 ± 0.225	1.66–3.40	2.83 ± 0.223	155.5	<0.001
Left	1.92–3.82	2.99 ± 0.288	2.05–3.82	3.15 ± 0.250	1.92–3.45	2.82 ± 0.221	149.6	<0.001
Average	2.14–3.81	2.99 ± 0.256	2.46–3.81	3.15 ± 0.204	2.14–3.36	2.83 ± 0.188	209.0	<0.001
LRA (cm)								
Right	0.26–1.14	0.72 ± 0.135	0.52–1.14	0.79 ± 0.116	0.26–0.96	0.65 ± 0.115	133.1	<0.001
Left	0.41–1.04	0.71 ± 0.127	0.46–1.04	0.77 ± 0.114	0.41–0.99	0.65 ± 0.106	99.58	<0.001
Average	0.41–1.09	0.71 ± 0.125	0.49–1.09	0.78 ± 0.109	0.41–0.93	0.65 ± 0.102	121.2	<0.001
MRA (cm)								
Right	0.74–1.52	1.11 ± 0.139	0.85–1.51	1.15 ± 0.127	0.74–1.52	1.06 ± 0.137	33.57	<0.001
Left	0.68–1.50	1.10 ± 0.139	0.82–1.50	1.15 ± 0.122	0.68–1.38	1.04 ± 0.135	50.34	<0.001
Average	0.72–1.48	1.10 ± 0.131	0.85–1.48	1.15 ± 0.118	0.72–1.38	1.05 ± 0.126	46.90	<0.001
C-P-LAP (cm)								
Right	0.34–2.82	1.86 ± 0.268	1.27–2.82	1.99 ± 0.233	0.34–2.31	1.72 ± 0.229	105.7	<0.001
Left	1.23–3.08	1.82 ± 0.252	1.43–3.08	1.95 ± 0.243	1.23–2.28	1.70 ± 0.192	98.10	<0.001
Average	0.92–2.56	1.84 ± 0.237	1.40–2.56	1.97 ± 0.211	0.92–2.15	1.71 ± 0.184	131.8	<0.001
C-P-MAP (cm)								
Right	1.40–3.64	2.65 ± 0.275	1.96–3.64	2.79 ± 0.237	1.40–3.13	2.51 ± 0.234	108.9	<0.001
Left	1.50–3.52	2.64 ± 0.282	1.80–3.52	2.79 ± 0.246	1.50–3.05	2.48 ± 0.230	123.4	<0.001

Average	1.78–3.50	2.64 ± 0.250	2.19–3.50	2.79 ± 0.205	1.78–3.05	2.49 ± 0.200	158.2	<0.001
C-LML (cm)								
Right	2.69–4.18	3.46 ± 0.329	3.31–4.18	3.73 ± 0.203	2.69–3.72	3.20 ± 0.186	555.8	<0.001
Left	2.84–4.30	3.52 ± 0.334	3.20–4.30	3.79 ± 0.207	2.84–3.91	3.26 ± 0.190	548.2	<0.001
Average	2.77–4.21	3.49 ± 0.323	3.32–4.21	3.76 ± 0.192	2.77–3.80	3.23 ± 0.173	641.8	<0.001
C-MML (cm)								
Right	2.90–4.30	3.75 ± 0.328	3.16–4.30	3.75 ± 0.240	2.90–3.98	3.26 ± 0.192	384.1	<0.001
Left	2.77–4.13	3.67 ± 0.322	3.08–4.13	3.67 ± 0.225	2.77–4.07	3.17 ± 0.187	426.0	<0.001
Average	2.85–4.17	3.71 ± 0.316	3.12–4.17	3.71 ± 0.221	2.85–4.03	3.21 ± 0.173	461.5	<0.001
MSI (cm)								
Right	1.81–3.56	2.81 ± 0.273	2.27–3.56	2.81 ± 0.255	1.81–3.42	2.56 ± 0.228	83.71	<0.001
Left	1.89–3.45	2.83 ± 0.288	2.02–3.45	2.83 ± 0.254	1.89–3.62	2.57 ± 0.259	78.38	<0.001
Average	1.93–3.51	2.82 ± 0.269	2.32–3.42	2.82 ± 0.244	1.93–3.51	2.56 ± 0.228	90.12	<0.001
LSI (cm)								
Right	1.68–3.52	2.76 ± 0.312	2.08–3.42	2.76 ± 0.278	1.68–3.52	2.48 ± 0.282	72.52	<0.001
Left	1.49–3.52	2.77 ± 0.305	2.20–3.45	2.77 ± 0.257	1.49–3.52	2.48 ± 0.280	88.45	<0.001
Average	1.67–3.40	2.76 ± 0.296	2.22–3.40	2.76 ± 0.256	1.67–3.28	2.48 ± 0.264	89.31	<0.001
MCW (cm)								
Right	2.06–3.57	2.90 ± 0.258	2.11–3.57	2.90 ± 0.222	2.06–3.42	2.61 ± 0.206	134.9	<0.001
Left	2.04–3.60	2.93 ± 0.284	2.17–3.60	2.93 ± 0.234	2.04–3.60	2.62 ± 0.242	126.9	<0.001
Average	2.11–3.55	2.92 ± 0.262	2.33–3.55	2.92 ± 0.219	2.11–3.51	2.62 ± 0.212	144.2	<0.001
LCW (cm)								
Right	1.51–3.52	2.56 ± 0.340	1.63–3.52	2.56 ± 0.292	1.51–3.41	2.21 ± 0.291	109.9	<0.001

Left	1.36–3.36	2.57 ± 0.329	1.96–3.35	2.57 ± 0.271	1.36–3.36	2.19 ± 0.270	145.1	<0.001
Average	1.50–3.35	2.56 ± 0.332	1.80–3.35	2.56 ± 0.267	1.50–3.15	2.20 ± 0.265	140.7	<0.001
MCMW (cm)								
Right	2.99–4.50	3.63 ± 0.331	3.31–4.50	3.93 ± 0.241	2.99–4.16	3.44 ± 0.201	366.3	<0.001
Left	2.89–4.48	3.61 ± 0.329	3.23–4.48	3.85 ± 0.229	2.89–4.24	3.37 ± 0.220	344.9	<0.001
Average	2.94–4.43	3.64 ± 0.322	3.27–4.43	3.89 ± 0.224	2.94–4.17	3.40 ± 0.198	397.4	<0.001
LCMW (cm)								
Right	2.63–4.37	3.50 ± 0.337	3.16–4.37	3.74 ± 0.253	2.63–3.83	3.26 ± 0.213	321.3	<0.001
Left	2.83–4.54	3.57 ± 0.328	3.18–4.40	3.78 ± 0.262	2.83–3.96	3.32 ± 0.203	340.9	<0.001
Average	2.77–4.40	3.53 ± 0.328	3.18–4.40	3.78 ± 0.244	2.77–3.79	3.29 ± 0.187	385.9	<0.001
FPL (cm)								
Right	0.00–1.96	0.80 ± 0.287	0.08–1.40	0.82 ± 0.241	0.00–1.96	0.78 ± 0.326	1.350	0.246
Left	0.17–2.26	0.87 ± 0.292	0.32–1.49	0.89 ± 0.227	0.17–2.26	0.86 ± 0.346	0.5707	0.450
Average	0.20–2.07	0.84 ± 0.267	0.20–1.40	0.85 ± 0.207	0.28–2.07	0.82 ± 0.315	1.081	0.299
LAEC (cm)								
Right	2.35–3.66	3.03 ± 0.291	2.73–3.66	3.24 ± 0.195	2.35–3.30	2.81 ± 0.190	385.0	<0.001
Left	2.38–3.80	3.11 ± 0.304	2.73–3.80	3.32 ± 0.209	2.38–3.53	2.89 ± 0.219	306.1	<0.001
Average	2.43–3.69	3.07 ± 0.288	2.78–3.69	3.28 ± 0.189	2.43–3.33	2.85 ± 0.189	395.1	<0.001
MAEC (cm)								
Right	1.90–3.62	2.75 ± 0.338	2.18–3.62	2.94 ± 0.284	1.90–3.28	2.56 ± 0.275	140.3	<0.001
Left	1.77–3.60	2.71 ± 0.321	2.15–3.60	2.89 ± 0.271	1.77–3.37	2.52 ± 0.256	145.5	<0.001
Average	1.84–3.61	2.73 ± 0.313	2.23–3.61	2.91 ± 0.260	1.84–3.33	2.54 ± 0.242	167.1	<0.001
AEML (cm)								

Right	0.63–2.46	1.71 ± 0.328	0.88–2.46	1.85 ± 0.280	0.63–2.32	1.56 ± 0.310	71.26	<0.001
Left	0.71–2.49	1.75 ± 0.338	0.85–1.61	1.89 ± 0.292	0.71–2.49	1.61 ± 0.322	63.01	<0.001
Average	1.84–3.61	1.73 ± 0.303	1.17–2.39	1.87 ± 0.253	0.67–2.32	1.59 ± 0.283	84.63	<0.001
AEMM (cm)								
Right	0.38–1.63	1.06 ± 0.282	0.49–1.63	1.16 ± 0.241	0.38–1.57	0.96 ± 0.287	40.56	<0.001
Left	0.42–1.66	1.10 ± 0.280	0.53–1.39	1.19 ± 0.228	0.42–1.66	1.00 ± 0.293	42.79	<0.001
Average	0.45–1.62	1.08 ± 0.260	0.58–1.62	1.17 ± 0.213	0.45–1.52	0.98 ± 0.267	49.57	<0.001
PCS (cm)								
Right	0.02–1.46	0.82 ± 0.296	0.40–1.46	0.89 ± 0.267	0.02–1.35	0.75 ± 0.306	18.72	<0.001
Left	0.01–1.48	0.86 ± 0.308	0.01–1.39	0.92 ± 0.293	0.01–1.48	0.80 ± 0.313	11.31	<0.001
Average	0.03–1.40	0.84 ± 0.275	0.21–1.40	0.90 ± 0.249	0.03–1.36	0.77 ± 0.284	17.79	<0.001
